# P-1815. Epidemiological Patterns of Dengue Fever in Children aged 0-14 years in Kathmandu, Nepal: A Seven-Year Analysis in a High-Altitude Setting (2018–2024)

**DOI:** 10.1093/ofid/ofaf695.1984

**Published:** 2026-01-11

**Authors:** Saugat Bhandari, Meeru Gurung, Sanjeev Man Bijukchhe, Bhishma Pokhrel, Puja Amatya, Anil Raj Ojha, Ruby Basi, Aayush Rizal, Rusy Shrestha, Shrijana Shrestha

**Affiliations:** Patan Academy of Health Sciences, Lalitpur Kathmandu, Bagmati, 44700Nepal, Bagmati, Nepal; Patan Academy of Health Sciences, Lalitpur Kathmandu, Bagmati, 44700Nepal, Bagmati, Nepal; Patan Academy of Health Sciences, Lalitpur Kathmandu, Bagmati, 44700Nepal, Bagmati, Nepal; Patan Academy of Health Sciences, Lalitpur Kathmandu, Bagmati, 44700Nepal, Bagmati, Nepal; Patan Academy of Health Sciences, Lalitpur Kathmandu, Bagmati, 44700Nepal, Bagmati, Nepal; Patan Academy of Health Sciences, Lalitpur Kathmandu, Bagmati, 44700Nepal, Bagmati, Nepal; Patan Academy of Health Sciences, Lalitpur Kathmandu, Bagmati, 44700Nepal, Bagmati, Nepal; Patan Academy of Health Sciences, Lalitpur Kathmandu, Bagmati, 44700Nepal, Bagmati, Nepal; Patan Academy of Health Sciences, Lalitpur Kathmandu, Bagmati, 44700Nepal, Bagmati, Nepal; Patan Academy of Health Sciences, Lalitpur Kathmandu, Bagmati, 44700Nepal, Bagmati, Nepal

## Abstract

**Background:**

Dengue fever is typically prevalent in low-lying, tropical regions. However, in recent years, cases have been observed in higher-altitude areas as well. The Kathmandu Valley, located at an elevation of approximately 1,400 meters above sea level, was previously considered a low-risk region for dengue. Despite this, an increasing number of paediatric cases have been documented in clinical settings. This study examines the trend of laboratory-confirmed non-structural protein 1 (NS1) positive dengue cases in children presenting to outpatient and emergency departments to a tertiary healthcare centre in Kathmandu over the past seven years.

**Methods:**

A retrospective review was conducted of all laboratory-confirmed NS1 positive dengue cases in children presenting to outpatient and emergency departments to Patan Hospital, a tertiary healthcare facility in Nepal, from 2018 to 2024. Data were collected on the number of cases per year and the gender distribution of patients to analyze trends and changes over time. A significant rise in cases was observed from 2019 onwards, coinciding with a major dengue outbreak in the Kathmandu Valley.

**Results:**

Between 2018 and 2024, a total of 857 paediatric dengue cases were recorded. Although no paediatric cases were reported cases in 2018, the number of cases began increasing in 2019 (33 cases) and surged sharply to a peak in 2022 (646 cases). Another noticeable increase was observed in early 2024 (150 cases). Male children were affected more than female children, with 528 cases among boys compared to 329 among girls. The majority of cases occurred during and after the monsoon season.

**Conclusion:**

Paediatric dengue cases have exhibited a rising trend in Kathmandu. Although the region was previously considered at low risk, the emerging threat now necessitates enhanced clinical vigilance and preparedness. Addressing the interplay between climate change and disease transmission is essential to mitigating future outbreaks and protecting paediatric populations in high-altitude settings.

**Disclosures:**

All Authors: No reported disclosures

